# Clinical Efficacy and Outcomes of Electro‐Pneumatic Intracorporeal Lithotripsy in the Management of Sialolithiasis

**DOI:** 10.1002/oto2.70080

**Published:** 2025-01-26

**Authors:** Iulian Filipov, Lucian Chirila, Mihai Sandulescu, Gheorghe Cristache, Corina Marilena Cristache

**Affiliations:** ^1^ Doctoral School “Carol Davila” University of Medicine and Pharmacy Bucharest Romania; ^2^ Department of Maxillofacial Surgery “Queen Maria” Military Emergency Hospital Brasov Romania; ^3^ Department of Oral and Maxillofacial Surgery “Carol Davila” University of Medicine and Pharmacy Bucharest Romania; ^4^ Department of Implant Prosthetic Therapy “Carol Davila” University of Medicine and Pharmacy Bucharest Romania; ^5^ Department of ENT Batistei Medical Center Bucharest Romania; ^6^ Department of Dental Techniques “Carol Davila” University of Medicine and Pharmacy Bucharest Romania

**Keywords:** calculus removal, electrohydraulic lithotripsy, intracorporeal lithotripsy, minimally invasive surgery, pneumatic lithotripsy, salivary gland obstruction, salivary gland stones, sialendoscopy, sialolithiasis

## Abstract

**Objective:**

This study aims to evaluate the clinical efficacy of electro‐pneumatic intracorporeal lithotripsy for the treatment of salivary gland stones.

**Study Design:**

A prospective cohort study of patients diagnosed with obstructive salivary gland syndrome, where basket‐assisted sialendoscopy alone failed to remove the calculi.

**Setting:**

This study was conducted at the “Queen Maria” Military Hospital in Brașov, Romania, and a private practice, between February 2023 and May 2024.

**Methods:**

A total of 29 patients with salivary calculi were treated using the SialoLither device (Hidromed), which operates on the electro‐pneumatic principle. The number of sessions required for complete stone removal, the duration of each session, and the number of ballistic impulses applied were recorded. Statistical analyses, including the Mann‐Whitney *U* test and multiple linear regression, were conducted to assess the relationship between stone size, location, and treatment outcomes.

**Results:**

Complete removal was achieved in 72.4% of patients after a single session, with 100% success after 3 sessions. The average number of impulses was 13.9 (±4.25), with no significant difference in outcomes between the parotid and submandibular glands (*P* > .05). The total duration varied, with a mean time of 89.97 (±54.89) minutes. Complications were minimal, with only 2 cases of minor epithelial damage.

**Conclusion:**

Electro‐pneumatic intracorporeal lithotripsy is a highly effective, minimally invasive technique for managing salivary calculi, offering a safe and efficient alternative to traditional surgical methods.

Obstructive salivary gland disease represents the most prevalent non‐neoplastic disorder affecting the major salivary glands.^1^ This condition is primarily characterized by recurrent episodes of pain and swelling, which often worsen during eating, and is caused by the presence of salivary calculi (stones) in 60% to 70% of cases.^2^, ^3^ Other contributing factors, such as ductal stenosis, mucous plugs, foreign bodies, and anatomical variations within the ductal system, may also play significant roles in the pathogenesis of this disorder.^2^

The pioneering development of salivary duct endoscopy, initiated in the late 1980s by Gundlach et al,^4^ Konigsberger et al,^5^ and Katz,^6^ marked a significant advancement in the field. Gundlach et al coined the term “sialendoscopy” and introduced it as a novel diagnostic procedure.^4^ Meanwhile, Konigsberger achieved the first successful application of endoscopically controlled laser lithotripsy (LL) in a patient with recurrent purulent sialadenitis of the left submandibular gland caused by sialolithiasis, demonstrating the therapeutic potential of this approach in the ENT field.^5^ Katz employed a 0.8‐mm flexible endoscope, offering a less invasive technique that permits direct intraluminal visualization and instrumentation of the salivary ductal system. This approach provides an effective alternative to the traditional method of salivary gland extirpation (sialadenectomy), which has been the standard definitive treatment for benign obstructive diseases of the major salivary glands.^3^

Despite the innovation of sialendoscopy, its effectiveness as a stand‐alone therapeutic method remains limited.^7^, ^8^, ^9^, ^10^, ^11^, ^12^ To enhance the outcomes of minimally invasive treatment strategies, various adjunctive techniques have been developed. Among these, intracorporeal sialolithotripsy, using either pneumatic or laser‐assisted methods,^13^ and extracorporeal shock wave lithotripsy (ESWL) are notable alternatives. These adjuvant approaches are particularly valuable in clinical scenarios where interventional sialendoscopy alone does not suffice to achieve the complete removal of salivary calculi.^10^, ^14^, ^15^, ^16^

Lithotripsy is a therapeutic procedure used to fragment sialoliths that cannot be directly removed with a basket due to their size or location. This fragmentation is achieved by applying physical forces generated by specialized equipment, making it easier to remove the calculi fragments either with the assistance of sialendoscopy or even spontaneously. Although both extracorporeal and intracorporeal lithotripsy aim to break down the calculi, these 2 methods differ significantly in terms of their mechanisms and procedural protocols.

However, the use of extracorporeal and intracorporeal lithotripsy in medical practice has significantly decreased the rate of salivary gland removal, reducing it from 40%‐50% to just 5%.^16^

Extracorporeal lithotripsy involves the use of external ultrasound waves generated by a sialolithotripter. The device's active component is placed against the skin overlying the affected gland, and ultrasound imaging is used to direct the shock waves toward the salivary calculus. These shock waves typically fragment the medium‐sized (≤5‐7 mm) and smaller sialoliths, facilitating their removal. The advantages of ESWL include its minimally invasive nature, the ability to perform the procedure in an outpatient setting with minimal patient discomfort, and a reduced recovery time. However, ESWL has certain limitations, such as the high cost of equipment and variable efficacy depending on the size, density, and location of the calculi. Additionally, one of the major constraints of ESWL is its limited availability, as the technology is accessible in only a few specialized centers, compounded by the fact that the machine is no longer in production. In many cases, multiple sessions and additional interventional sialendoscopy procedures are necessary. ESWL is particularly suitable for patients with parotid gland sialolithiasis, where the success rate is significantly higher compared to its use for submandibular gland calculi.^10^, ^14^, ^15^ This observation is well supported by evidence in the literature, which attributes the difference to several key factors.^14^, ^15^, ^17^ Anatomical differences play a significant role, as the parotid duct is straighter and less convoluted than the submandibular duct, facilitating more efficient transmission of shock waves to the target stone and reducing the likelihood of shock wave energy dispersion. Additionally, stone composition is a crucial factor; parotid stones are often less dense and primarily phosphate‐based, making them more amenable to fragmentation with ESWL. In contrast, submandibular stones tend to be denser and are often composed of calcium oxalate or carbonate, which makes them more resistant to fragmentation.^18^

Unlike extracorporeal lithotripsy, endoscopically assisted intraductal shock wave lithotripsy (ISWL), which has been recently introduced into medical practice for the treatment of obstructive salivary gland pathology, utilizes pneumatic, laser, or electrohydraulic techniques with miniature probes.^19^ These probes are inserted into the salivary duct through a sialendoscope and make direct contact with the sialoliths.^20^ This method, particularly when using pneumatic or laser assistance, is suitable for medium‐sized sialoliths (measuring between 5 and 7 mm).^21^ The success of this therapeutic approach depends on several factors, including visibility, the degree of stone mobility, the size and shape of the sialolith, its location, and its relationship to the salivary duct.^22^, ^23^

Endoscopically assisted ISWL is a minimally invasive therapeutic option that has demonstrated good outcomes in published studies.^16^, ^19^, ^21^, ^24^ Compared to ESWL, ISWL offers several advantages, including the use of less expensive equipment, direct visualization of the calculus for more precise targeting, and more efficient transmission of shock waves (pneumatic, laser, or electrohydraulic) to the sialolith. Significant advancements have been achieved through the development of specialized sialendoscopes, instruments, and devices. Both LL and intraductal pneumatic lithotripsy (IPL) used in ISWL have shown success rates of 90% or higher.^13^, ^22^, ^25^, ^26^ However, fewer studies have been conducted on electro‐pneumatic lithotripsy,^20^, ^27^ making its efficacy less well‐documented. The aim of the present study was to specifically evaluate electro‐pneumatic lithotripsy by assessing its success based on various outcomes, including the time required to completely remove calculi for each patient, the number of ballistic impulses needed for fragmentation, the number of sessions required for stone removal, and any intraoperative and postoperative complications.

## Materials and Methods

This prospective study was conducted at the “Queen Maria” Military Hospital in Brașov, Romania, as well as in a private practice setting. The study adhered to the ethical standards outlined in the World Medical Association's Declaration of Helsinki, the Belmont Report, the Council for International Organizations of Medical Sciences (CIOMS) guidelines, and the International Conference on Harmonization Good Clinical Practice (ICH‐GCP) standards. The approval was granted by the Ethics Committee of the “Carol Davila” University of Medicine and Pharmacy (approval number 19772/2023), and all participants provided informed consent.

Between February 2023 and May 2024, all patients diagnosed with obstructive salivary gland syndrome, in whom calculi could not be removed using basket‐assisted sialendoscopy, were included in this cohort study. The cohort consisted of consecutive patients meeting the predefined inclusion criteria. Exclusion criteria encompassed stones that were not fully visible under sialendoscopy or were located in inaccessible areas, such as deep intraparenchymal stones. Patients whose salivary calculi were successfully removed using basket‐assisted sialendoscopy or who had contraindications to sialendoscopy or lithotripsy were also excluded. Contraindications included severe ductal strictures, extensive intraparenchymal involvement deemed unsuitable for minimally invasive interventions, or other clinical conditions precluding the safe application of these procedures.

Ultrasonography, conducted in all cases using the Siemens Acuson Juniper (Siemens Healthineers AG), served as the primary diagnostic tool and was instrumental in guiding the therapeutic approach for each patient. Performed by a surgeon with significant training and expertise in ultrasonography, it was also utilized at the conclusion of each session to confirm the presence or absence of residual stones, ensuring comprehensive evaluation and procedural accuracy.

**Figure 1:**
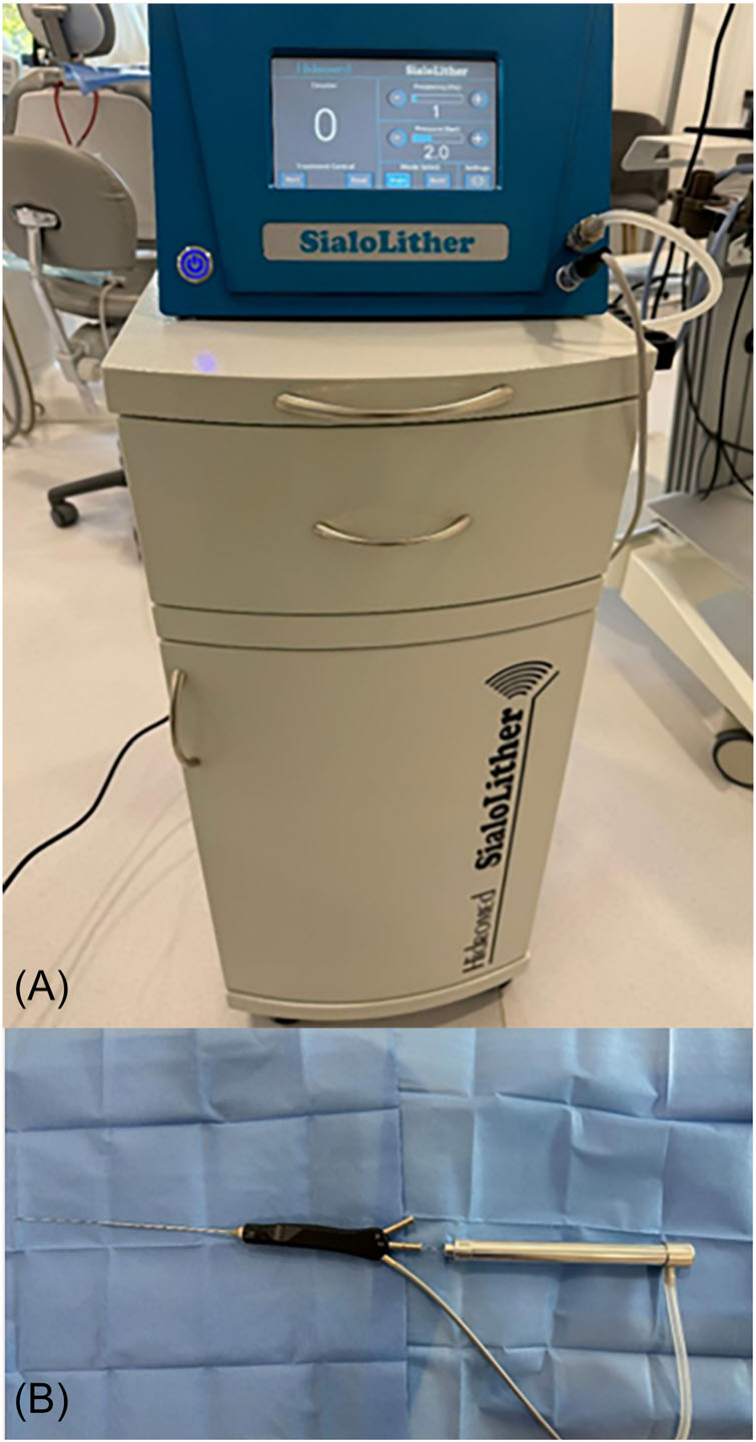
SialoLither lithotripter: (A) central unit and trolley incorporating the compressed air cylinder; (B) 1.6‐mm “all‐in‐one” sialendoscope (Karl Storz) and 0.7‐mm probe.

The treatment for the patients included in this study was carried out under local anesthesia, using the SialoLither (Hidromed), a device specifically designed for the treatment of obstructive salivary gland pathology, particularly for fragmenting sialoliths (Figure 1A). The device is certified by the European Commission (EC) according to the Medical Device Regulation for the treatment of salivary stones and operates on the electro‐pneumatic principle. The SialoLither uses probes with diameters of 0.6 mm and 0.7 mm, allowing them to fit into the 0.8‐mm working channel of the 1.6‐mm “all‐in‐one” Karl Storz sialendoscope (Figure 1B). The lithotripter consists of a handheld instrument to which the probe is attached, and it is activated by ballistic energy generated from a compressed air cylinder. The electro‐pneumatic energy fragments the sialolith without damaging the surrounding soft tissue. Since the system relies solely on ballistic impacts from compressed air, no heat is generated that could potentially harm the salivary duct epithelium or the endoscope, eliminating the risk of thermal injury.

The following describes the technique used for intracorporeal lithotripsy assisted by pneumatic methods. First, the salivary duct orifice was identified using a flexible 0.015‐inch wire (Cook Medical), which was inserted 0.5 to 1 cm into the Wharton or Stensen duct. A 22 G cannula was then used to access the salivary duct, and a syringe filled with anesthetic was attached to flush anesthetic along the duct. After removing the cannula, the orifice of the Stensen or Wharton duct was dilated using a conical dilator or a series of dilators starting from 0.5 mm, allowing the sialendoscope to be introduced. The sialendoscopes used were “all‐in‐one” models (Karl Storz) with a diameter of 1.6 mm. Once the orifice was sufficiently dilated, the sialendoscope was inserted into the duct, and, under continuous irrigation with 0.9% sodium chloride, the duct was explored until the sialolith was located. If the stone's size, shape, or position made removal with a basket impossible, the lithotripter was employed.

The lithotripter's 0.7‐mm probe was inserted into the 0.8‐mm working channel, and the device was set to an intensity of 2.5 bars, with 1 shock per pedal activation. Once the probe was visualized and direct contact with the sialolith was established, pneumatic impulses were applied to the stone by pressing the pedal. A variable number of impulses were administered, with periodic saline flushing to aid in the process. Careful handling and continuous flushing ensured accurate fragmentation while minimizing trauma to the salivary duct epithelium. When fragmentation and mobilization of the stone fragments were observed, the probe was removed, and a basket was introduced into the working channel to capture and remove the larger fragments. After thorough saline flushing and removal of the sialendoscope, manual pressure was applied to the gland to help evacuate smaller fragments through the natural salivary flow.

The main variables measured in the study included the patient's age, gender, the size and position of the sialolith(s), the time required to completely remove the calculi, the number of ballistic impulses needed for fragmentation, the number of sessions required for stone removal, and any intraoperative or postoperative complications.

The treatment was considered successful if the sialolith(s) were completely removed. Follow‐up evaluations were conducted through periodic check‐ups at 7 days, 3 weeks, and 2 to 3 months post‐treatment. These assessments involved mandatory local clinical examinations for the first 2 follow‐ups, and, when necessary, follow‐up by phone depending on the patient's availability.

### Statistical Analysis

The collected data were entered into an Excel document and analyzed using IBM® SPSS® statistical software, version 25.0 (IBM Corp.). Descriptive statistics were calculated for numerical values, including percentage, mean, and standard deviation (SD). The Mann‐Whitney *U* test was employed to compare treatment times and impulses between the parotid and submandibular glands. To evaluate the influence of calculus size and location on the total time and total impulses required for its complete removal, a multiple linear regression analysis was conducted. For cases where more than 1 session was needed, the treatment time and number of impulses were summed across the required sessions. The statistical significance was set at *P* < .05.

## Results

**Figure 2:**
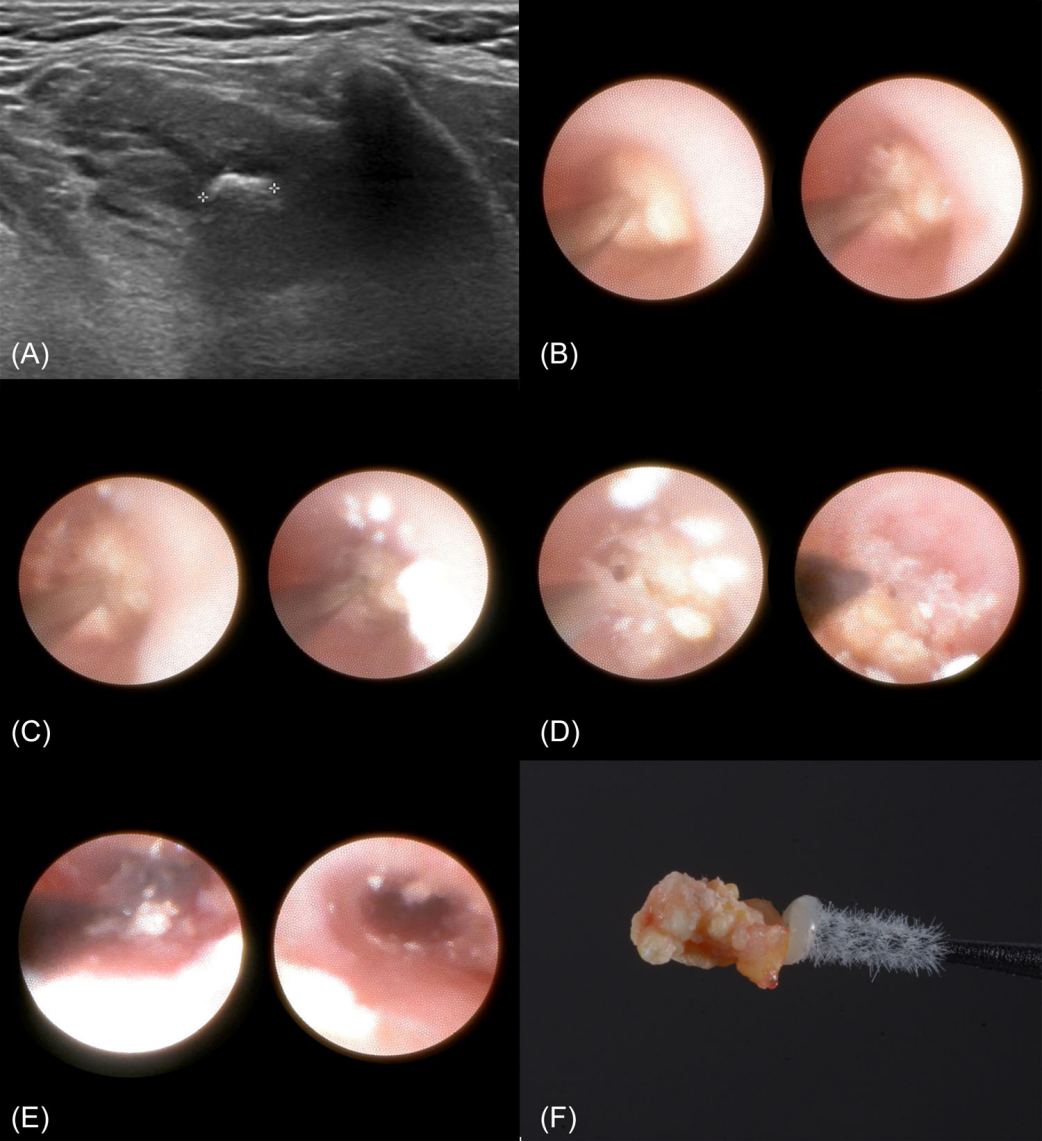
A 3.1/5.2 mm calculus in the left submandibular gland. (A) Ultrasonography image; (B‐E) sequential endoscopic images during sialolithotripsy; (F) photograph of the removed calculus.

This cohort study included 29 participants, consisting of 18 women (62%) and 11 men (38%), aged between 16 and 78 years, with a mean age of 45.10 years. The analysis of the sialolith localization revealed that 17 cases (58.6%) had stones within the Wharton's duct of the submandibular gland, while the remaining 12 cases (41.4%) involved sialoliths in the Stensen's duct of the parotid gland. The majority of the stones were located in the hilar region (27/29 cases), while 2 were intraparenchymal in the submandibular gland. An example of the procedure for a case involving a 3.1 × 5.2 mm calculus in the Wharton's duct of a left submandibular gland is presented in Figure 2.

For cases where stones were easily palpable and fixed, a direct basket‐assisted sialendoscopy approach was initially attempted, aligning with the study protocol's emphasis on prioritizing minimally invasive techniques. Lithotripsy was introduced only when basket‐assisted removal proved unsuccessful due to factors such as the size, location, or immobility of the calculi. This stepwise approach ensured that lithotripsy was reserved for complex cases where simpler methods were inadequate.

Intracorporeal sialolithotripsy was employed as an adjunct procedure alongside conventional interventional sialendoscopy in all cases.

Sialolithotomy was not necessary in any of the cases, as larger fragments were successfully extracted through basket‐assisted sialendoscopy following lithotripsy.

Ultrasound was routinely conducted after each session to confirm the absence of residual stones.

Successful fragmentation of the sialoliths in a single session was achieved in 21 cases (72.4%), while 6 cases (20.6%) required 2 sessions for complete removal. In 2 cases (6.8%), 3 sessions were necessary to fully fragment and remove the stones. The number of ballistic impulses applied during the procedures ranged from 6 to 23, with a mean of 13.9 (±4.25), reflecting the varying complexities and resilience of the sialoliths.

The duration of the intracorporeal interventions ranged from 24 to 218 minutes (with the maximum time spread across 3 sessions), with a mean duration of 89.97 (±54.89) minutes. This wide range indicates that some cases were straightforward, requiring minimal time, while others were more complex, necessitating extended procedures. The average size of the sialoliths, as measured by ultrasonography, was 5.67 mm (±1.54), showing a range of stone sizes treated during the study.

There was no significant difference in treatment time (*P* = .53) or impulses (*P* = .88) between the parotid and submandibular glands. The size of the sialoliths had a small positive relationship with both total time and total impulses, but these relationships were not statistically significant. Similarly, the location coefficient indicated that parotid procedures took slightly more time and required slightly more impulses than submandibular ones, but this difference was also not statistically significant. The *R*‐squared value for total time was 0.036, meaning that only 3.6% of the variation in total time was explained by calculus size and location, which is quite low. For total impulses, the *R*‐squared value was even lower at 0.004 (0.4%), indicating that size and location contributed very little to the variation in total impulses. Neither the size nor the location of the sialoliths had a statistically significant effect on the total time or impulses required for removal (Table 1).

**Table 1:** Results of Multiple Linear Regression Analysis on the Influence of Calculus Size and Location on the Total Time and Total Impulses Required for Its Complete Removal

	Coefficient		*P* value		
Dependent variable	Size	Location	Size	Location	*R* ^2^
Total impulses	2.02	19.96	.77	.35	0.035
Total time	0.11	0.47	.88	.78	0.004

Complications were minimal but notable. In 2 cases, minor epithelial scraping lesions were observed in the salivary ducts, which were associated with mild pain during postoperative evaluation, likely due to procedural trauma. At the first follow‐up, 1 week after the procedure, 23 of the 29 patients reported being completely symptom‐free. The remaining 6 patients experienced mild, transient symptoms, either occurring spontaneously or triggered by meals, likely due to residual swelling or mild epithelial trauma caused by the endoscope or basket manipulation. These symptoms did not indicate any severe or permanent injury.

By the 3‐week follow‐up and again at 2 to 3 months post‐operation, all patients reported a complete absence of symptoms. This excellent recovery rate highlights the efficacy and safety of the combined approach of conventional sialendoscopy and intracorporeal sialolithotripsy in treating sialolithiasis, with minimal invasiveness and swift postoperative recovery in the majority of cases.

## Discussion

Obstructive sialadenitis caused by salivary stones is a challenging condition that significantly impacts patients' quality of life through recurrent pain and inflammation.^28^, ^29^ Traditional surgical approaches are associated with high morbidity risks, including infections, nerve damage, and prolonged recovery.^30^, ^31^ Less invasive alternatives, such as endoscopically assisted sialolithotomy via an oral approach or sialolithotomy using a transfacial approach, are considered the gold standard in current practice.^32^, ^33^, ^34^ However, despite their minimally invasive nature, these procedures still carry risks of damaging surrounding anatomical structures. In recent years, the introduction of minimally invasive techniques, particularly intracorporeal sialolithotripsy, has revolutionized the management of sialolithiasis by offering a less invasive method of stone fragmentation, reducing recovery time and minimizing risks.

Laser‐assisted lithotripsy has been widely adopted, with the Holmium YAG (Ho:YAG) laser being one of the most commonly used methods.^20^, ^21^ However, LL poses potential risks, as 40% of the laser's energy can be reflected off the stone surface, possibly causing damage to the salivary duct epithelium.^16^ Despite these limitations, intracorporeal lithotripsy with laser technology has reported success rates between 81% and 100%.^16^ Recent studies^20^ have shown that pneumatic lithotripters, such as Vibrolith (ELMED Medical Systems), which is primarily approved for renal and urinary tract stones but certified for salivary stone use, offer comparable results. Koch et al^20^ demonstrated a success rate of up to 98.7% using pneumatic lithotripsy, although their study required a higher number of ballistic impulses (mean 366.54 ± 39.86 impulses per stone) compared to laser treatments. Furthermore, the average procedure time reported by Koch et al^20^ was shorter (50.96 ± 3.42 minutes) than that in other studies.

In contrast, our study, the first to report on the use of an electro‐pneumatic lithotripter specifically designed for salivary stones in Europe, achieved a 100% success rate, with 72.4% of cases completed in a single session and all remaining cases resolved within 3 sessions. The mean number of ballistic impulses in our study was significantly lower (mean 13.9 ± 4.25 impulses), aligning more closely with the results of Serbetci et al,^27^ who reported an average of 13 impulses (range 6‐36) using similar pneumatic methods. Our procedure times were longer (mean 89.97 ± 54.89 minutes) compared to those reported by Koch et al,^20^ likely due to procedural complexity, though parotid gland cases took longer on average, without statistical significance. The focus of our study was to evaluate the feasibility and efficacy of electro‐pneumatic lithotripsy as an adjunctive technique in cases where basket‐assisted sialendoscopy alone proved insufficient. Lithotripsy is not intended to replace sialolithotomy but to complement it in specific clinical scenarios, such as with stones that are too large or fixed to be removed directly. In this context, intracorporeal lithotripsy was employed even for stones located in the canal or hilum of the gland, including those that were palpable and accessible through simpler techniques like sialolithotomy, to prioritize preserving the integrity of the duct and surrounding structures. This study contributes to existing knowledge by demonstrating the efficacy and safety of electro‐pneumatic lithotripsy in salivary gland stone removal, offering a novel, minimally invasive alternative to laser and pneumatic lithotripters. Unlike laser‐based methods, the residual energy from the electro‐pneumatic approach does not affect the salivary duct epithelium, reducing the risk of tissue damage. Additionally, our findings underscore that the procedure can achieve excellent results with fewer impulses and a low complication rate. Only 2 cases of minor epithelial scraping were observed, resulting in mild, transient postoperative symptoms, with complete symptom resolution in all patients within 3 weeks.

In summary, this study reinforces the utility of electro‐pneumatic‐assisted intracorporeal sialolithotripsy as a highly effective and minimally invasive treatment for sialolithiasis. It provides an important addition to the literature by highlighting an alternative that combines safety, efficacy, and low complication rates, particularly for cases where direct basket‐assisted sialendoscopy fails. Despite the small cohort, our study demonstrates the potential for broader application of electro‐pneumatic lithotripsy in clinical practice.

One limitation of our study is the relatively small sample size, which may reduce the statistical power to detect significant differences between groups. This limits the generalizability of our findings and suggests that further studies with larger cohorts are needed to confirm the results.

## Conclusions

Despite its limitations, this study demonstrates the effectiveness and safety of electro‐pneumatic‐assisted intracorporeal lithotripsy for the removal of salivary calculi, achieving complete fragmentation in up to 3 sessions. The technique allows precise control over calculus fragmentation without causing thermal damage or tissue complications, thus preserving salivary gland function. An operating pressure of 2.5 bars was consistently used, and no major complications were observed. This method shows significant potential as a minimally invasive alternative to more traditional, invasive surgical procedures. This study underscores the importance of incorporating advanced lithotripsy technologies to offer patients safer and more effective treatments for sialolithiasis.

## Author Contributions

**Iulian Filipov**, surgery, substantial contributions to the conception of the work, drafting the work, final approval of the version to be published, agreement to be accountable for all aspects of the work in ensuring that questions related to the accuracy or integrity of any part of the work are appropriately investigated and resolved; **Lucian Chirila**, substantial contributions to the data acquisition, drafting the work, final approval of the version to be published, agreement to be accountable for all aspects of the work in ensuring that questions related to the accuracy or integrity of any part of the work are appropriately investigated and resolved; **Mihai Sandulescu**, substantial contributions to the interpretation of data for the work, reviewing it critically for important intellectual content, final approval of the version to be published, agreement to be accountable for all aspects of the work in ensuring that questions related to the accuracy or integrity of any part of the work are appropriately investigated and resolved; **Gheorghe Cristache**, substantial contributions to the design of the work and data acquisition, drafting the work, final approval of the version to be published, agreement to be accountable for all aspects of the work in ensuring that questions related to the accuracy or integrity of any part of the work are appropriately investigated and resolved; **Corina Marilena Cristache**, substantial contributions to the design of the work, analysis and interpretation of data for the work, reviewing it critically for important intellectual content, final approval of the version to be published, agreement to be accountable for all aspects of the work in ensuring that questions related to the accuracy or integrity of any part of the work are appropriately investigated and resolved.

## Disclosures

### Competing interests

The authors declare that there is no conflict of interest.

### Funding source

This research did not receive any specific grant from funding agencies in the public, commercial, or not‐for‐profit sectors.
